# Isolation and characterization of diverse antimicrobial lipopeptides produced by *Citrobacter* and *Enterobacter*

**DOI:** 10.1186/1471-2180-13-152

**Published:** 2013-07-08

**Authors:** Santi M Mandal, Shalley Sharma, Anil Kumar Pinnaka, Annu Kumari, Suresh Korpole

**Affiliations:** 1 Central Research Facility, Indian Institute of Technology Kharagpur, Kharagpur-721302, West Bengal, India; 2 MTCC and Gene Bank, CSIR-Institute of Microbial Technology, Sector 39A, Chandigarh 160036, India

**Keywords:** *Citrobacter*, *Enterobacter*, Antimicrobial lipopeptide, MALDI and phylogenetic analysis

## Abstract

**Background:**

Increasing multidrug-resistance in bacteria resulted in a greater need to find alternative antimicrobial substances that can be used for clinical applications or preservation of food and dairy products. Research on antimicrobial peptides including lipopeptides exhibiting both narrow and broad spectrum inhibition activities is increasing in the recent past. Therefore, the present study was aimed at isolation and characterization of antimicrobial lipopeptide producing bacterial strains from fecal contaminated soil sample.

**Results:**

The phenotypic and 16S rRNA gene sequence analysis of all isolates identified them as different species of Gram-negative genera *Citrobacter* and *Enterobacter*. They exhibited common phenotypic traits like citrate utilization, oxidase negative and facultative anaerobic growth. The HPLC analysis of solvent extracts obtained from cell free fermented broth revealed the presence of multiple antimicrobial lipopeptides. The comprehensive mass spectral analysis (MALDI-TOF MS and GC-MS) of HPLC purified fractions of different isolates revealed that the lipopeptides varied in their molecular weight between (m/z) 607.21 to 1536.16 Da. Isomers of mass ion m/z 984/985 Da was produced by all strains. The 1495 Da lipopeptides produced by strains S-3 and S-11 were fengycin analogues and most active against all strains. While amino acid analysis of lipopeptides suggested most of them had similar composition as in iturins, fengycins, kurstakins and surfactins, differences in their β-hydroxy fatty acid content proposed them to be isoforms of these lipopeptides.

**Conclusion:**

Although antimicrobial producing strains can be used as biocontrol agents in food preservation, strains with ability to produce multiple antimicrobial lipopeptides have potential applications in biotechnology sectors such as pharmaceutical and cosmetic industry. This is the first report on antibacterial lipopeptides production by strains of *Citrobacter* and *Enterobacter*.

## Background

Various species of genera like *Clostridium*, *Escherichia*, *Listeria*, *Salmonella, Shigella, Staphylococcus* and *Vibrio*[1,2] are known to cause food spoilage. In addition, different drug resistant strains of *Escherichia* and *Salmonella* belonging to family *Enterobacteriaceae* are reported to cause food-borne illness [3-6]. Increasing multidrug-resistance in bacteria resulted in a greater need to find alternative antimicrobial substances that can be used for various applications including clinical as well as preservation of food and dairy products. Therefore, research on antimicrobial peptides including antimicrobial biosurfactants as a new class of drugs has increased in the recent past as they exhibit both narrow and broad spectrum inhibition activities against Gram-positive and Gram-negative bacteria or fungi. Although members of the *Enterobacteriaceae* family are known to produce bacteriocins such as enterocins by *Enterobacter* sp. [7], serracin by *Serratia* sp. [8] bacteriocin by *Citrobacter* sp. [9] and microcins by *Escherichia* sp. [10], they are not reported to produce any antimicrobial biosurfactants. The different types of biosurfactants with antimicrobial activity include lipopeptides, glycolipids, phospholipids and lipopolysaccharides [11]. While many Gram-positive bacteria including different species of the genus *Bacillus* are reported to produce diverse antimicrobial lipopeptides with different applications in pharmaceutical and food processing industries [12], only few lipopeptides have been reported to produce by Gram-negative bacteria like *Pseudomonas*[13]. The lipopeptides produced by Gram-positive strains have been classified into various types based on their amino acid composition and fatty acid chain length [14]. Similarly, lipopeptides of *Pseudomonas* also have been grouped into different groups including amphisin, syringomycin, tolaasin and viscosin based on the number and composition of amino acids [13,15,16]. Among the several types of biosurfactants, lipopeptides belonging to iturins [17], surfactins, [18], fengycins [19], kurstakins [20], bacillomycins [21] and mycosubtilin [22] displayed therapeutic applications [23] and they were never reported to produce by any Gram-negative bacteria. Therefore, in the present study we have isolated few Gram-negative bacterial strains belonging to genera *Citrobacter* and *Enterobacter* producing antimicrobial lipopeptides from a fecal contaminated soil sample. Further, detailed characterization of these antimicrobial lipopeptides assigned them to iturins, fengycins, kurstakins and surfactins, usually produced by Gram-positive bacteria.

## Results

### Identification of the lipopeptide producing strains

**Figure 1:**
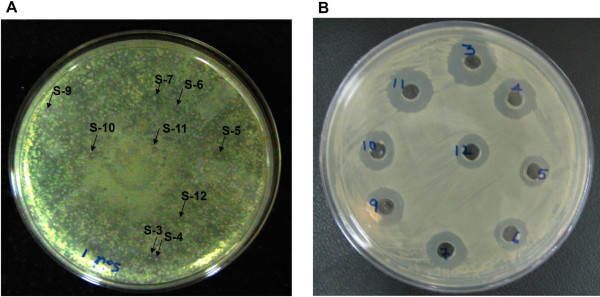
**Screening of isolates for antimicrobial activity. (A)** colonies showing zone of clearance **(B)** well diffusion assay of methanol extracts. Selected colonies were purified and preserved. Further, methanol extracts were prepared from 48 h cell free fermented broth of all selected isolates and tested against *S. aureus* (MTCC1430).

**Figure 2:**
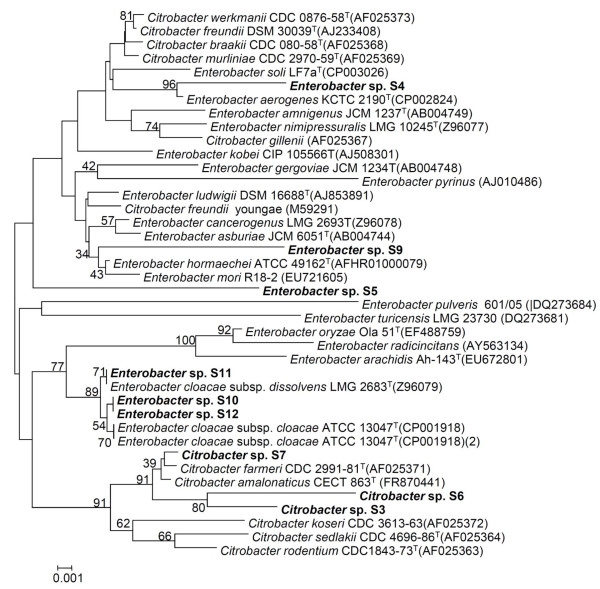
**Neighbour-joining phylogenetic tree of 16S rRNA gene sequences of all strains showing the relationship with members of the genera *Citrobacter *and *Enterobacter*.** The tree is drawn to scale, with branch lengths in the same units as those of the evolutionary distances used to infer the phylogenetic tree.

Nine antimicrobial producing strains were isolated from a fecal contaminated soil sample during a screen to isolate the bacteriocin producing bacteria. The colonies were selected based on colony morphology and the zone of clearance in their surroundings that might be formed due to the activity of antimicrobial substances produced by the strain (Figure 1A). The isolates grew well on tryptone soya agar (TSA) between pH 5.0 to 9.0 and up to 42°C temperature with optimum growth at 37°C. All strains were rod shaped, facultative anaerobes, showed positive reaction to catalase and negative for oxidase activities. The 16S rRNA gene sequence BLAST analysis revealed high identity with *Citrobacter farmeri* for strains S-3, S-6 and S-7. Other strains including S-4, S-5 and S-9 had identity with different species of the genus *Enterobacter*. Strains S-10, S-11 and S-12 showed high similarity with *E. cloacae* subsp. *dissolvens*. Further, Phylogenetic analysis with close relatives also assigned them to genera *Citrobacter* and *Enterobacter* of the family *Enterobacteriaceae*. In neighbour-joining phylogenetic tree, strains S-3, S-6 and S-7 formed a cluster with *C. farmeri* and *C. amalonaticus* (Figure 2). Although isolate S-9 showed 98.1% identity with *E. mori* in 16S rRNA gene blast analysis, it formed an out group to the clade containing *E. hormaechei* and *E. mori* with low bootstrap value. Overall, most of the clusters of the neighbour-joining phylogenetic tree showed low bootstrap values.

### Isolation and antimicrobial activity of lipopeptides

**Figure 3:**
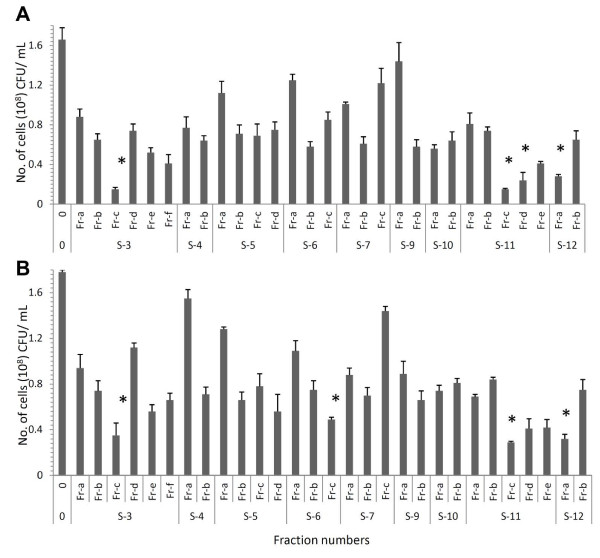
**Determination of antibacterial property of lipopeptide fractions.** The assay performed against Gram positive *S. epidermidis***(A)** and Gram negative *P. aeruginosa***(B)** bacteria. Data are the means calculated from three replicate experiments and vertical bars correspond to standard deviations. Asterisk represents significant differences between treatments and negative control (0) with p<0.005 using one-way ANOVA followed by Dunnett’s test. The results are presented as the mean of triplicates (n=3) ± SD.

The methanol extracts of lipopeptides obtained from different strains (mentioned as sample S-3 to S-12) were tested for antimicrobial activity using *Staphylococcus aureus* (MTCC1430) as test strain (Figure 1B) and subsequently purified using RP-HPLC. Methanol extract of each sample showed multiple peaks during their HPLC analysis and the number of peaks differed for individual strain. The extract obtained from strain S-3 yielded a maximum number of six peaks followed by strains S-11 and S-5. Individual lipopeptides (fractions) collected from extracts of different strains were purified and used to find their antimicrobial activity against Gram-positive and Gram-negative test strains. Though, *S. epidermidis* (MTCC435) and *Pseudomonas aeruginosa* (ATCC27853) were taken as representative Gram-positive and Gram-negative indicator strains initially, subsequently antimicrobial activity was tested against *S. aureus*, *Micrococcus luteus* (MTCC106) and *Candida albicans* (MTCC1637). Majority of fractions showed activity towards Gram-positive indicator strains (Figure 3A) and variations observed in relative sensitivity of Gram-negative test strain towards different antimicrobial lipopeptide fractions (Figure 3B). Overall, lipopeptide fractions obtained from strains S-3 and S-11 showed highest activity against test strains. In particular, fractions Fr-c and Fr-e of strain S-11 exhibited maximum antimicrobial activity against *S. aureus* and *M. luteus* at lower concentrations by inhibiting the complete growth, however, none of the lipopeptides inhibited the growth of yeast like *C. albicans* (data not shown).

### Determination of minimum inhibitory concentration (MIC) and sensitivity

The MIC analysis of purified lipopeptide fraction Fr-c of strain S-11 revealed 12, 15 and 16 μg/ml concentration for Gram-positive test strains *M. luteus*, *S. aureus* and *S. epidermidis*, respectively. In contrast, Gram-negative test strains like *Serratia marcescens* and *P. aeruginosa* exhibited MIC of 20 and 32 μg/ml respectively. Results of heat stability assay of these lipopeptides fractions revealed no reduction in activity even after exposing to temperature of 100°C for 30 min (data not shown).

### Mass spectrometry characterization of lipopeptides

**Figure 4:**
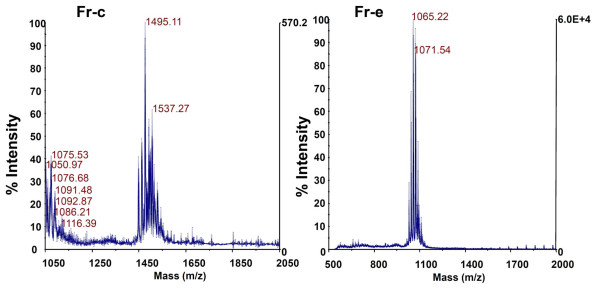
MALDI MS spectrum of Fr-c and Fr-e from strainS-3 (identical spectrum is observed with the Fr-c and Fr-e of the strain S-11).

The HPLC purified individual lipopeptide fractions were collected, confirmed their purity by reinjection into HPLC and used for the structure determination by MALDI-TOF mass spectrometry. Results of analysis of all HPLC fractions revealed the presence of various lipopeptide species. The mass ion with m/z 984/985 Da was observed in fractions of lipopeptides produced by all strains (Table 1) and the GC MS analysis for fatty acid identification suggested that it had a β-hydroxylated C_15_ fatty acid. Additional GC-MS analysis of all HPLC purified fractions documented the presence of β-hydroxy fatty acid with a chain length from C_7_ to C_17_. The fractions Fr-c and Fr-e found commonly in strains S-3 and S-11 showed high antimicrobial activity and the molecular mass determined for these lipopeptides were m/z 1495 and 1065, respectively (Figure 4). The fatty acid analysis revealed that fractions Fr-c and Fr-e contained β-hydroxy fatty acids with chain lengths C_17_ and C_14_ respectively, suggesting that these compounds belong to the antimicrobial lipopeptide family fengycin and iturin respectively. Further, the amino acid sequence obtained for the fraction Fr-c (EOrnYTEVPEYV) confirmed it as a member of fengycin family. The molecular mass and fatty acid composition of fraction with m/z 1043 (Fr-a of sample S-6) assigned it to lipopeptide group surfactin. Other antimicrobial mass ions produced by these strains include m/z 607, 637, 679, 721, 746, 1153, 1180, 1522 and 1535.

**Table 1:** List of masses observed in fractionated lipopeptides from different samples obtained in positive ion linear mode

**Sample name**	**HPLC fraction number**	**Mass (m/z)**	**SD**
Sample S-3	Fr-a	985.13	0.0021
	Fr-b	985.73	0.0037
	Fr-c	1495.11	0.0069
	Fr-d	1522.52	0.003
	Fr-e	1065.22	0.0034
	Fr-f	607.21	0.01
Sample S-4	Fr-a	679.57	0.0052
	Fr-b	984.82	0.01
Sample S-5	Fr-a	679.69	0.0092
	Fr-b	984.77	0.01
	Fr-c	637.06	0.05
	Fr-d	746.17	0.0042
Sample S-6	Fr-a	1043.66	0.01
	Fr-b	984.96	0.0059
	Fr-c	637.01	0.0071
Sample S-7	Fr-a	1180.01	0.022
	Fr-b	985.01	0.015
	Fr-c	721.25	0.0011
Sample S-9	Fr-a	1536.16	0.0092
	Fr-b	984.57	0.01
Sample S-10	Fr-a	1535.21	0.0074
	Fr-b	984.21	0.0098
Sample S-11	Fr-a	1153.65	0.0075
	Fr-b	984.22	0.0012
	Fr-c	1495.43	0.0045
	Fr-d	637.23	0.025
	Fr-e	1065.21	0.01
Sample S-12	Fr-a	679.23	0.003
	Fr-b	984.14	0.0091

The calculations of standard deviation (SD) were done using MS Excel Descriptive Statistics for each ion measurements (n=4), mi is the measured mass and following is the formula: $\sqrt{\frac{{\sum_{f}\left(m_{1}-{\bar{m}}_{1}\right)}^{2}}{n-1}}$.

## Discussion

Several reports have described that soil microbes are worthy to be used as the source of different antimicrobial substances including peptides for versatile applications [24]. Most of the antimicrobial peptides produced by diverse bacteria inhibit Gram-positive strains like *Listeria monocytogens* but not Gram-negative [25]. Among the various peptides, lipopeptides are well known to inhibit the growth of fungi and bacteria including opportunistic pathogens. Consequently, naturally produced antimicrobial lipopeptides have been receiving increased attention due to their anti-infective nature with wide antimicrobial spectrum. Besides the activity of natural peptides, any chemical modifications in structure of these lipopeptide are shown to improve their spectrum and activity. To this effect, daptomycin, an anionic lipopeptide has already been used for therapeutic applications [26]. While antimicrobial lipopeptides are produced by different Gram-positive and Gram-negative bacteria, only lipopeptides produced by species of *Pseudomonas* and *Bacillus* have been studied in detail [13,14,27-29]. In the present study several antimicrobial substances producing bacterial strains were isolated from a fecal contaminated soil sample and characterization of these substances revealed them as antimicrobial lipopeptides.

The phenotypic features like Gram-negative staining, catalase positive, oxidase negative, facultative anaerobic growth and citrate utilization observed for all strains suggested that they belong to the *Enterobacteriaceae* family, usually observed in fecal matter. The 16S rRNA gene sequence blast analysis and subsequent phylogentic analysis assigned all strains to different species of the genera *Citrobacter* and *Enterobacter*. Interestingly, though strains S-5 and S-9 displayed high identity with *E. hormaechei* and *E. mori* respectively in 16S rRNA gene sequence, they only formed an out group to the cluster comprised of different *Enterobacter* and *Citrobacter* species (Figure 2). However, the overall topology of neighbour-joining tree revealed the phylogenetic complexity and discrepancies in 16S rRNA gene sequences of strains belonging to the family *Enterobacteriaceae*. It was also supported by the unusual inclusion of different species belonging to genera *Citrobacter* and *Enterobacter* in the same cluster suggesting the need to revisit the family *Enterobacteriaceae*.

The antimicrobial lipopeptides typically contain a cyclic or linear oligopeptide linked with a β-hydroxy fatty acid tail of varied lengths [28]. Inhibition spectra of these lipopeptides are influenced by the composition of oligopeptide as well as fatty acid component [30,31]. Antimicrobial lipopeptides are largely produced by Gram-positive bacteria like *Bacillus* sp. and are classified into different families based on the composition of oligopeptides and antibacterial or antifungal activities [32]. Among the Gram-negative bacteria, *Pseudomonas* is the only genus reported to produce antimicrobial lipopeptides such as massetolide, viscosin [33], syringomycin [34], arthrofactin [35], pseudodesmins [36], orfamide [16] and putisolvin [37]. In addition to these lipopeptides, species like *P. fluorescens* was reported to produce different massetolide analogues [33]. However, lipopeptides produced by Gram-positive or Gram-negative bacteria had different composition and so far no bacterial strain has been reported to produce reciprocal combinations of lipopeptide composition. Therefore, in the present study we made an attempt to characterize lipopeptides produced by the strains of genera *Citrobacter* and *Enterobacter*.

The comprehensive mass spectral (MALDI-TOF MS and GC-MS) analysis of HPLC purified antimicrobial lipopeptides obtained from strains of *Citrobacter* and *Enterobacter* revealed the occurrence of different lipopeptide antibiotics belonging to groups like kurstakin, iturin, surfactin and fengycin, usually produced by Gram-positive bacteria. Further, individual lipopeptide belonging to a particular group shown to exhibit differences in their amino acids [13,27], fatty acid chain length or isomers of fatty acids and thus generating various analogues with varied activity [13,33]. Accordingly, lipopeptides of the present study showed differences in fatty acid composition and also differed in their antibacterial activity. Of the various lipopeptides, the lipopeptide fraction Fr-b produced by all strains had a molecular weight of 984/985 Da. Although amino acid composition of this peptide identified it as kurstakin, it differed in fatty acid composition (C_15_) when compared to other kurstakin members that contained fatty acids with chain length of C_11_-C_14_, suggesting the lipopeptide fraction (Fr-b) is an isoform of kurstakin. Further, differences in antimicrobial activity spectrum of these peptides attributed to the fatty acid composition differences [20].

A variety of lipopeptides produced by strains *Citrobacter* sp. strain S-3 and *Enterobacter* sp. strain S-11 were identified as lipopeptides belonging to iturin, kurstakin and fengycin with unusual broad spectrum antibacterial activity. It is pertinent to mention that the fraction Fr-e of strains S-3 and S-11, had an identical mass with the lipopeptide reported by Swart and Merwe [38], therefore, we have minimized further attempt to characterize the full sequence as reported [β-NC_14_NYNQPNS]. Additionally, identification of C_14_ fatty acid as the lipid content of the fraction Fr-e also confirmed their classification under iturins as they are known to contain a fatty acid chain length of C_14_ to C_16_[39] along with a cyclic peptide of seven amino acids. Cyclic lipopeptide biosurfactants like iturin, mycosubtilin, surfactin and kurstakin are largely produced by species of *Bacillus* exhibiting antimicrobial activity [12,28]. In fact, iturin and fengycin produced by *B. subtilis* are recognized as potential biopharmaceutical agents due to their antimicrobial and biosurfactant properties [14]. Although different types of lipopeptides varied in their amino acid and/or fatty acid composition, they all are usually thermostable, resistant to proteolytic enzymes and inhibits the growth by altering the membrane integrity. Similarly no reduction in antibacterial activity was observed for fraction Fr-e upon exposing it to 100°C temperature for 30 min suggesting it as heat stable. The amino acid composition analysis of highly active lipopeptide fraction (Fr-c) of strain S-3 and S-11 revealed the sequence as R(C17)EOrnYTEVPEYV which corresponds to linearized fengycin B’2, an isoform produced by a *B. subtilis* strain [40]. Among the other lipopeptide fractions, Fr-f (m/z 607.21 Da) and Fr-d (m/z 637.23 Da), produced by strains S-3 and S-11, respectively, showed significant antimicrobial activity, but could not be assigned to any lipopeptide family as their molecular mass did not match with any reported antimicrobial lipopeptides. Other mass ions, except m/z 679 Da, produced by different strains did not show significant antimicrobial activity against any test strain. Although iturins, kurstakins, surfactins and fengycins differed in composition, they followed the same mechanisms such as involving pore formation on bacterial membrane [41] or by other non-specific interactions with the membrane [42] as a result of their antimicrobial activity. Findings of this study, together with the fact that the entire isolated strains belong to *Citrobacter* or *Enterobacter* and antimicrobial lipopeptide production ability, suggests that they are possibly produced by these bacteria as a part of defence mechanism to survive in complex environments.

## Conclusions

This is the first report on antibacterial lipopeptides production by strains of *Citrobacter* and *Enterobacter* that are part of the human intestinal flora and frequently observed in food. The lipopeptides are exceedingly useful molecules with potential applications in several biotechnology sectors such as pharmaceutical, cosmetic, preservation of food and dairy products. However, engineering of these molecules is very important for our future needs as the large scale production of antimicrobial lipopeptides is expensive. Therefore, strains like S-3 or S-11 with ability to co-produce different antimicrobial lipopeptides are very useful in biotechnology sector. Increased lipopeptides production by these strains through the optimization of physicochemical parameters or transcriptional regulation of lipopeptide synthetase gene clusters could be future insight for commercial production.

## Methods

### Isolation of bacteria and identification

The bacterial isolates designated as S-3, S-4, S-5, S-6, S-7, S-9, S-10, S-11 and S-12 were isolated from a fecal contaminated soil sample. The soil sample used to isolate the strains was serially diluted and plated on nutrient agar with the following composition (g/l): peptic digest of animal tissue, 5.0; beef extract, 1.5; yeast extract, 1.5; sodium chloride, 5.0; agar 15.0 (pH adjusted to 7.2). Colonies with inhibition zone in their surroundings were selected and streaked on to fresh nutrient agar (NA, HiMedia, India) medium plates. Upon testing their purity all isolates were preserved at -70°C for further studies. Indicator strains used to test the activity of lipopeptides were obtained from Microbial Type Culture Collection and Genebank (MTCC), Chandigarh, India. The test strains were grown on tryptone soya agar (TSA) medium with the following composition (g/l): pancreatic digest of casein, 15.0; papaic digest of soybean meal, 5.0; sodium chloride, 5.0; agar 15.0 and the pH adjusted to 7.2. All isolates producing antimicrobial lipopeptides were tested for phenotypic properties including morphology, physiology and biochemical characteristics using standard procedures. The identity of isolates was also confirmed by using 16S rRNA gene sequence [43] blast search analysis. All 16S rRNA gene sequences of the nearest type strains were downloaded from the NCBI database and aligned using CLUSTAL_W program of MEGA version 5 [44]. The alignment was corrected manually using the BioEdit sequence alignment editor [45]. Pair-wise evolutionary distances were calculated with the Kimura two-parameter [46] and a neighbour-joining phylogenetic tree was constructed using the MEGA version5.0. The stability of phylogenetic tree was assessed by taking 1000 replicates. All sequences have been submitted to EMBL database [accession nos. HF572835 - HF572843].

### Extraction of lipopeptides

Lipopeptides produced by all strains were isolated from culture supernatant by a combination of acid and solvent extraction procedure [47]. In brief, cells were pellet down from the culture broth by centrifugation (13,000 × g) for 15 min at 4°C. The supernatant pH was adjusted to 2.0 by addition of concentrated HCl and allowed to precipitate at 4°C for 16 h. After centrifugation (13,000 × g) for 20 min at 4°C the precipitate was collected and extracted with methanol by stirring for 2 h. The lipopeptide containing methanol was collected after filtration and vacuum-dried.

### Purification of lipopeptides

The lipopeptides extracted were dissolved in methanol and fractionated by reverse phase- HPLC (Agilent 1100 series, CA, USA) with a ZORBAX 300-SB18 column (4.6 mm × 250 mm, particle size 5 μm), at a flow rate of 1 ml/min. The solvent system used was (A) 0.1% aqueous TFA and (B) acetonitrile containing 0.1% TFA. The following gradient of solvent B was used to run the column: 0-60% for 0-45 min, 60-80% for 45-55 min and 80-100% for 55-60 min. All peptides eluted from the column were monitored at 215 nm in a diode array detector and all peaks obtained during HPLC were collected using a fraction collector (GILSON, France) that is coupled with the system. These fractions were concentrated by speed vacuum and tested for their antimicrobial activity. The fractions or peaks that showed antibacterial activity were re-chromatographed in the same column under similar conditions, except solvent B was used as 100% acetonitrile with a gradient of 0-10% for 30 min. The peptide concentration was determined using the RP-HPLC conditions and calibrated with surfactin (Sigma-Aldrich, St. Louis, USA).

### Antimicrobial assay and MIC determination

All fractions from the reversed phase HPLC were lyophilized, redissolved in MilliQ water to test their antimicrobial activity against *S. epidermidis* (MTCC435) and *P. aeruginosa* (ATCC27853) in a microtiter plate assay in triplicates. To examine the bacterial growth or killing rate in the presence of different fractions, bacterial cells were grown in 100 μl of Mueller-Hinton broth (MHB, HiMedia, India) supplemented with fixed concentration (10 μg/ml) of each fraction, at 37°C. Growth or killing rates were determined by measuring OD at 600 nm. The OD values were converted into concentration of cells measured in CFU per millilitre (1.0 OD corresponded to 2.16 × 10^8^ CFU/ml). The MIC of selected biosurfactant/lipopeptide was evaluated for strains *S. aureus* (MTCC1430), *M. luteus* (MTCC106) and *S. marcescens* (MTCC 97) along with *P*. *aeruginosa* and *S. epidermidis* by using a microtiter plate dilution assay in triplicates as described earlier [48]. Test strains were grown to logarithmic phase (between 0.3-0.4 OD) under optimal conditions. The lowest concentration inhibiting the growth of test strain without showing any increase in absorption up to 48 h of incubation was considered as MIC.

### MALDI-TOF-MS and sequencing

The purified and active lipopeptides were analysed for molecular mass and MS/MS sequencing by using a Voyager time-of-flight mass spectrometer (Applied Biosystems, Foster City, CA, USA). For MS/MS sequencing, the lactone ring present in lipopeptide was cleaved by incubating each peptide with 10% NaOH in methanol at room temperature for 16 h. The cleaved peptide obtained was lyophilized and again extracted with methanol, and allowed for mass spectrometry analysis. Spectra were recorded in the post-source decay (PSD) ion mode as an average of 100 laser shots with a grid voltage of 75%. The reflector voltage was reduced in 25% steps and guide wire was reduced 0.02–0.01% with an extraction delay time of 100 ns.

### Fatty acid analysis by GC-MS

To analyze the fatty acid content associated with the lipopeptides, the peptides (5 mg of each) were incubated with 0.5 ml of 6 M HCl at 90°C for 18 h in sealed tubes for acid hydrolysis. The fatty acids were extracted with ether, treated with 0.95 ml methanol and 0.05 ml of 98% H_2_SO_4_ at 65°C for 6 h. Finally, fatty acid methyl esters were obtained with n-hexane extraction and analyzed on GC-MS with a Clarus 500 GC (PerkinElmer, USA). The carrier gas used was helium with a flow rate of 1.0 ml/min. The column temperature was maintained at 120°C for 3 min and thereafter gradually increased (8°C/min) to 260°C.

### Statistical analysis

The statistical significance of the experimental results was determined using one-way ANOVA followed by Dunnett’s test. Values of p<0.05 were considered statistically significant. Prism version 5.0 was used for all statistical analyses. The results are presented as the mean of triplicates (n=3) ± SD.

## Competing interests

The authors declare that they have no competing interests.

## Authors’ contributions

SMM and SS isolated the strains and performed experiments involving identification and characterization of strains and lipopeptides, antimicrobial activity assay, analysed the data and wrote the paper. AKP performed the phylogenetic analysis of the strains. AK participated in 16S rRNA gene sequencing and phenotypic characterization of isolates. SK participated in the design, coordination of experiments, analysis of the data and writing the manuscript. All authors read the final manuscript and approved the same.

## References

[B1] Graveland H Wagenaar JA Heesterbeek H Mevius D van Duijkeren E Heederik D Methicillin resistant *Staphylococcus aureus* ST398 in veal calf farming: human MRSA carriage related with animal antimicrobial usage and farm hygiene PLoS ONE 2010 5 6 e10990 10.1371/journal.pone.0010990 20544020 PMC2882326

[B2] Vanderhaeghen W Hermans K Haesebrouck F Butaye P Methicillin-resistant *Staphylococcus aureus* (MRSA) in food production animals Epidemiol Infect 2010 138 5 606 625 10.1017/S0950268809991567 20122300

[B3] Gorman R Adley CC Characterization of *Salmonella enterica* serotype *Typhimurium* isolates from human, food, and animal sources in the Republic of Ireland J Clin Microbiol 2004 42 5 2314 2316 10.1128/JCM.42.5.2314-2316.2004 15131222 PMC404650

[B4] Hammerum AM Heuer OE Human health hazards from antimicrobial-resistant *Escherichia coli* of animal origin Clin Infect Dis 2009 48 7 916 921 10.1086/597292 19231979

[B5] Vidovic S Korber DR Prevalence of *Escherichia coli* O157 in Saskatchewan cattle: characterization of isolates by using random amplified polymorphic DNA PCR, antibiotic resistance profiles, and pathogenicity determinants Appl Environ Microbiol 2006 72 6 4347 4355 10.1128/AEM.02791-05 16751550 PMC1489585

[B6] Zhao S White DG Friedman SL Glenn A Blickenstaff K Ayers SL Abbott JW Hall-Robinson E McDermott PF Antimicrobial resistance in *Salmonella enteric* serovar *Heidelberg* isolates from retail meats, including poultry, from 2002 to 2006 Appl Environ Microbiol 2008 74 21 6656 6662 10.1128/AEM.01249-08 18757574 PMC2576681

[B7] de Graaf FK Tieze GA Wendelaar Bonga S Stouthamer AH Purification and genetic determination of bacteriocin production in *Enterobacter cloacae* J Bacteriol 1968 95 2 631 640 4867749 10.1128/jb.95.2.631-640.1968 PMC252062

[B8] Jabrane A Sabri A Compere P Jacques P Vandenberghe I Van Beeumen J Thonart P Characterization of serracin P, a phage-tail-like bacteriocin, and its activity against *Erwinia amylovora*, the fire blight pathogen Appl Environ Microbiol 2002 68 11 5704 5710 10.1128/AEM.68.11.5704-5710.2002 12406768 PMC129874

[B9] Shanks RMQ Dashiff A Alster JS Kadouri DE Isolation and identification of a bacteriocin with antibacterial and antibiofilm activity from *Citrobacter freundii* Arch Microbiol 2012 194 7 575 587 10.1007/s00203-012-0793-2 22290290 PMC3408838

[B10] Chiuchiolo MJ Delgado MA Farias RN Salomon RA Growth-phase-dependent expression of the cyclopeptide antibiotic microcin J25 J Bacteriol 2001 183 5 1755 1764 10.1128/JB.183.5.1755-1764.2001 11160108 PMC95062

[B11] Parkinson M Biosurfactants Biotechnol Adv 1985 3 1 65 83 10.1016/0734-9750(85)90006-0 14541779

[B12] Rodrigues L Banat IM Teixeira J Oliveira R Biosurfactants: potential applications in medicine J Antimicrob Chemother 2006 57 4 609 618 10.1093/jac/dkl024 16469849

[B13] Nybroe O Sørensen J Ramos J-L Production of cyclic lipopeptides by fluorescent pseudomonads Pseudomonas, Biosynthesis of Macromolecules and Molecular Metabolism 2004 New York: Kluwer Academic/Plenum Publishers 147 172

[B14] Ongena M Jacques P *Bacillus* lipopeptides: versatile weapons for plant disease biocontrol Trends Microbiol 2008 16 3 115 125 10.1016/j.tim.2007.12.009 18289856

[B15] Bender CL Scholz-Schroeder BK Ramos J-L New insights into the biosynthesis, mode of action and regulation of syringomycin, syringopeptin and coronatine Pseudomonas Vol2, Virulence and Gene Regulation Volume 2 2004 New York: Kluwer Academic/Plenum Publishers 125 158

[B16] Gross H Loper JE Genomics of secondary metabolite production by *Pseudomonas* spp Nat Prod Rep 2009 26 11 1408 1446 10.1039/b817075b 19844639

[B17] Delcambe L Peypoux F Besson F Guinand M Michel G Structure of iturin-like substances Biochem Soc Trans 1977 5 1122 1124 913800 10.1042/bst0051122

[B18] Arima K Kakinuma A Tamura G Surfactin, a crystalline peptide lipid surfactant produced by *Bacillus subtilis*: isolation, characterization and its inhibition of fibrin clot formation Biochem Biophys Res Commun 1968 31 3 488 494 10.1016/0006-291X(68)90503-2 4968234

[B19] Vanittanakom N Loeffler W Koch U Jung G Fengycin- a novel antifungal lipopeptide antibiotic produced by *Bacillus subtilis* F-29-3 J Antibiot 1986 39 7 888 901 10.7164/antibiotics.39.888 3093430

[B20] Hathout Y Ho Y-P Ryzhov V Demirev P Fenselau C Kurstakins: a new class of lipopeptides isolated from *Bacillus thuringiensis* J Nat Prod 2000 63 11 1492 1496 10.1021/np000169q 11087590

[B21] Roongsawang N Thaniyavarn J Thaniyavarn S Kameyama T Haruki M Imanaka T Morikawa M Kanaya S Isolation and characterization of halotolerant *Bacillus subtilis* BBK-1 which produces three kinds of lipopeptides: bacillomycin L, plipastatin and surfactin Extremophiles 2002 6 6 499 506 10.1007/s00792-002-0287-2 12486459

[B22] Duitman HE Hamoen LW Rembold M Venema G Seitz H Saenger W Bernhard F Reinhard R Schmidt M Ullrich C Stein T Leenders F Vater J The mycosubtilin synthetase of *Bacillus subtilis* ATCC6633: A multifunctional hybrid between a peptide synthetase, an amino transferase and a fatty acid synthase Proc Natl Acad Sci USA 1999 96 23 13294 13299 10.1073/pnas.96.23.13294 10557314 PMC23941

[B23] Besson F Michel G Biosynthesis of iturin and surfactin by *Bacillus subtilis*: evidence for amino acid activating enzymes Biotechnol Lett 1992 14 11 1013 1018 10.1007/BF01021050 1644198

[B24] Mandal SM Barbosa AE Franco OL Lipopeptides in microbial infection control: scope and reality for industry Biotechnol Adv 2013 (In press), S0734-9750(13)00006-2. 10.1016/j.biotechadv.2013.01.004 23318669

[B25] Abee T Krockel L Hill C Bacteriocins: modes of action and potentials in food preservation and control of food poisoning Int J Food Microbiol 1995 28 2 169 185 10.1016/0168-1605(95)00055-0 8750665

[B26] Tally FP De Bruin MF Development of daptomycin for Gram-positive infections J Antimicrob Chemother 2000 46 4 523 526 10.1093/jac/46.4.523 11020247

[B27] Baindara P Mandal SM Chawla N Singh PK Pinnaka AK Korpole S Characterization of two antimicrobial peptides produced by a halotolerant *Bacillus subtilis* strain SK.DU.4 isolated from a rhizosphere soil sample AMB Express 2013 3 1 2 10.1186/2191-0855-3-2 23289832 PMC3549917

[B28] Raaijmakers JM De Bruijn I Nybroe O Ongena M Natural functions of lipopeptides from *Bacillus* and *Pseudomonas*: more than surfactants and antibiotics FEMS Microbiol Rev 2010 34 6 1037 1062 20412310 10.1111/j.1574-6976.2010.00221.x

[B29] Raaijmakers JM De Bruijn I de Kock JD Cyclic lipopeptide production by plant-associated *Pseudomonas* spp. diversity, activity, biosynthesis and regulation Mol Plant Microbe Interact 2006 19 7 699 710 10.1094/MPMI-19-0699 16838783

[B30] Jerala R Synthetic lipopeptides: a novel class of antiinfectives Expert Opin Investig Drugs 2007 16 8 1159 1169 10.1517/13543784.16.8.1159 17685866

[B31] Makovitzki A Avrahami D Shai Y Ultrashort antibacterial and antifungal lipopeptides Proc Natl Acad Sci USA 2006 103 43 15997 16002 10.1073/pnas.0606129103 17038500 PMC1635116

[B32] Price NPJ Rooney AP Swezey JL Perry E Cohan FM Mass spectrometric analysis of lipopeptides from *Bacillus* strains isolated from diverse geographical locations FEMS Microbiol Lett 2007 271 1 83 89 10.1111/j.1574-6968.2007.00702.x 17419767

[B33] De Bruijn I de Kock MJ de Waard P van Beek TA Raaijmakers JM Massetolide A biosynthesis in *Pseudomonas fluorescens* J Bacteriol 2008 190 8 2777 2789 10.1128/JB.01563-07 17993540 PMC2293227

[B34] Dumenyo CK Mukherjee A Chun W Chatterjee AK Genetic and physiological evidence for the production of N-acyl homoserine lactones by *Pseudomonas syringae* pv. *syringae* and other fluorescent plant pathogenic *Pseudomonas* species Eur J Plant Pathol 1998 104 6 569 582 10.1023/A:1008651300599

[B35] Roongsawang N Hase K Haruki M Imanaka T Morikawa M Kanaya S Cloning and characterization of the gene cluster encoding arthrofactin synthetase from *Pseudomonas* sp. MIS38 Chem Biol 2003 10 9 869 880 10.1016/j.chembiol.2003.09.004 14522057

[B36] Sinnaeve D Michaux C Van Hemel J Vandenkerckhove J Peys E Borremans FAM Sas B Wouters J Martins JC Structure and X-ray conformation of pseudodesmins A and B, two new cyclic lipodepsipeptides from *Pseudomonas* bacteria Tetrahedron 2009 65 21 4173 4181 10.1016/j.tet.2009.03.045

[B37] Dubern JF Lugtenberg BJ Bloemberg GV The ppulrsaL-ppuR quorum-sensing system regulates biofilm formation of *Pseudomonas putida* PCL1445 by controlling biosynthesis of the cyclic lipopeptides putisolvins I and II J Bacteriol 2006 188 8 2898 2906 10.1128/JB.188.8.2898-2906.2006 16585751 PMC1447005

[B38] Swart MRP van der Merwe MJ Sequence specific stabilization of a linear analog of the antifungal lipopeptide iturin A2 by sodium during low energy electrospray ionization mass spectrometry conditions J Am Soc Mass Spectrom 2001 12 5 505 516 10.1016/S1044-0305(01)00232-X 11349948

[B39] Hourdou ML Besson F Tenoux I Michel G Fatty acids and β-amino acid syntheses in strains of *Bacillus subtilis* producing iturinic antibiotics Lipids 1989 24 11 940 944 10.1007/BF02544538 2515402

[B40] Pathak KV Keharia H Gupta K Thankur SS Balaram P Lipopeptides from the Banyan endophyte, *Bacillus subtilis* K1: mass spectrometric characterization of a library of fengycins J Am Soc Mass Spectrom 2012 23 10 1716 1728 10.1007/s13361-012-0437-4 22847390

[B41] Deleu M Paquot M Nylander T Fengycin interaction with lipid monolayers at the air-aqueous interface implications for the effect of fengycin on biological membranes J Colloid Interface Sci 2005 283 2 358 365 10.1016/j.jcis.2004.09.036 15721905

[B42] Bessalle R Kapitkovsky A Gorea A Shalit I Fridkin M All-D-magainin: chirality, antimicrobial activity and proteolytic resistance FEBS Lett 1990 274 1–2 151 155 2253768 10.1016/0014-5793(90)81351-n

[B43] Suresh K Mayilraj S Chakrabarti T *Effluviibacter roseus* gen. nov. sp. nov., isolated from muddy water, belonging to the family “*Flexibacteraceae*” Int J Syst Evol Microbiol 2006 56 7 1703 1707 10.1099/ijs.0.64144-0 16825654

[B44] Tamura K Peterson D Peterson N Stecher G Nei M Kumar S MEGA5: molecular evolutionary genetics analysis using maximum likelihood, evolutionary distance and maximum parsimony methods Mol Biol Evol 2011 28 10 2731 2739 10.1093/molbev/msr121 21546353 PMC3203626

[B45] Hall TA BioEdit: a user-friendly biological sequence alignment editor and analysis program for Windows 95/98/NT Nucleic Acids Symp Ser 1999 41 95 98

[B46] Kimura M A simple method for estimating evolutionary rates of base substitutions through comparative studies of nucleotide sequences J Mol Evol 1980 16 2 111 120 10.1007/BF01731581 7463489

[B47] Vater J Kablitz B Wilde C Franke P Mehta N Cameotra SS Matrix-assisted laser desorbtion ionization-time of flight mass spectrometry of lipopeptide biosurfactants in whole cells and culture filtrates of *Bacillus subtilis* C-1 isolated from petroleum sludge Appl Environ Microbiol 2002 68 12 6210 6219 10.1128/AEM.68.12.6210-6219.2002 12450846 PMC134408

[B48] Singh PK Chittpurna A Sharma V Patil PB Suresh K Identification, purification and characterization of laterosporulin, a novel bacteriocin produced by *Brevibacillus* sp. Strain GI-9 PLoS ONE 2012 7 3 e31498 10.1371/journal.pone.0031498 22403615 PMC3293901

